# The Pathogenesis of Atopic Dermatitis‐Trends for the Future

**DOI:** 10.1111/1346-8138.70164

**Published:** 2026-02-03

**Authors:** Norito Katoh

**Affiliations:** ^1^ Kyoto Prefectural University of Medicine Kyoto Japan

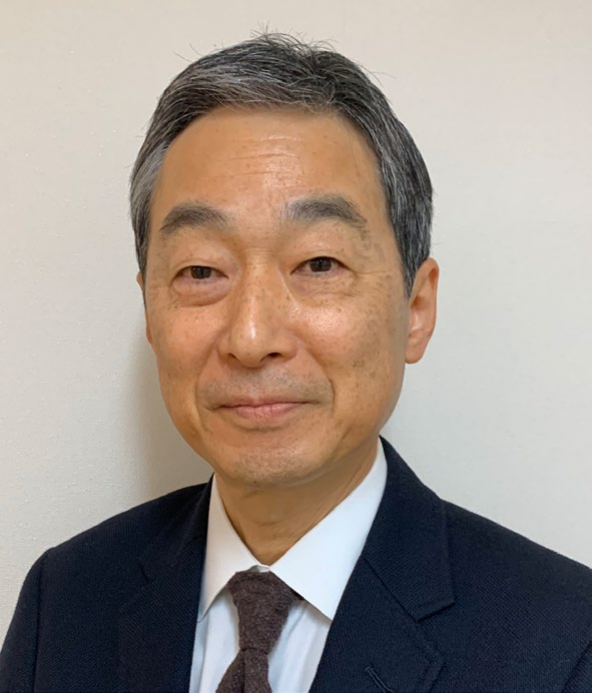

Atopic dermatitis (AD) was formerly regarded as a chronic inflammatory skin disease with an ambiguous pathogenesis. However, recent advances in science and technology have elucidated the complex pathogenesis of AD. The primary findings indicate that two abnormalities, “skin barrier dysfunction” typified by filaggrin gene mutations, and “type 2 inflammation” centered on IL‐4 and IL‐13, interact to form the pathogenesis of AD. Sakai introduces the latest findings on the vicious cycle between type 2 inflammation and stratum corneum lipid abnormalities and the utility of noninvasive assessment of stratum corneum ceramides as biomarkers of disease activity, therapeutic response, and relapse risk.

It has long been known that *Staphylococcus aureus* is frequently detected in AD lesions and has been considered one of the exacerbating factors. Okamoto and Matsuoka provide the current knowledge on functional dysbiosis and the accessory gene regulator quorum‐sensing system in the pathogenesis of AD, and discuss the future therapeutic applications. Antimicrobial peptides work as regulators of immune responses and skin barrier homeostasis in addition to innate defenders against microbial invasion including bacteria, fungus, and virus. Peng and Niyonsaba et al. describe the recent findings about antimicrobial peptides in the pathogenesis of AD and emerging therapeutic strategies.

Advances in research into the pathogenesis of AD have brought many new therapeutic options to patients who were previously unable to induce remission. Given the heterogeneity of AD, it is hypothesized that the involvement of each pathogenic factor is subject to variation among individual patients. It is, therefore, important to understand the mechanisms of action of each treatment for AD and to select the optimal therapy for individual patients in daily clinical practice. I hope that this special issue will help readers understand future trends in the pathogenesis of AD.

## Conflicts of Interest

Norito Katoh has received honoraria as a speaker/consultant for Sanofi, Maruho, Abbvie, Ely‐Lilly Japan, Taiho Pharmaceutical, Pfizer, Mitsubishi Tanabe Pharma, Jansen Pharma, Kyowa Kirin, Celgene Japan, Torii Pharmaceutical, Novartis Pharma, and Otsuka Pharmaceutical and has received grants as an investigator from Mitsubishi Tanabe Pharma, Torii Pharmaceutical, Maruho, Sun Pharma, Boehringer Ingelheim Japan, and Leo Pharma.

## Data Availability

The data that support the findings of this study are available upon request from the corresponding author. The data are not publicly available due to privacy or ethical restrictions.

